# Social Sustainability in Aging Populations: A Systematic Literature Review

**DOI:** 10.1093/geront/gnad097

**Published:** 2023-08-01

**Authors:** Kathrin Komp-Leukkunen, Juho Sarasma

**Affiliations:** Department of Social Sciences, LUT University, Lappeenranta, Etelä-Karjala, Finland; Faculty of Social Sciences, University of Helsinki, Helsinki, Uusimaa, Finland; Fortum Oyj, Helsinki, Uusimaa, Finland

**Keywords:** Aging societies, Demographic change, Intergenerational justice, Well-being

## Abstract

**Background and Objectives:**

Social sustainability becomes increasingly important in aging populations. Yet, scientific discussions on this topic are still emerging. This study helps to develop these discussions by exploring (1) how social sustainability is understood in studies on aging populations, (2) how this understanding differs across the topics discussed, and (3) how population aging is connected to social sustainability.

**Research Design and Methods:**

This study conducts a systematic literature review using 33 texts obtained from ProQuest, JStor, and scholar.google.com (02–03/2022). The inclusion criteria were (1) scientific character and (2) explicit focus on the topics of interest. A thematic analysis was conducted.

**Results:**

The texts use 3 different understandings of social sustainability: one focusing on what makes societies desirable, one focusing on the quality of life of individuals, and one balancing the interests of current and future generations. The first understanding is most prevalent. Which understanding texts choose depends on their topic, perspective, and goals. The texts describe challenges and opportunities for social sustainability in aging populations, with some recommending a general shift in perspective.

**Discussion and Implications:**

Findings provide a clearer and more homogenous understanding of social sustainability for discussions on population aging. Thereby, they facilitate a dialogue between researchers working in this area. Moreover, they help gerontologists increase their contribution to cumulative knowledge building. A limitation is that only texts in English are analyzed. Findings help policymakers and practitioners better understand how to integrate research-based knowledge on social sustainability in their work.

## Background and Objectives

Sustainability is at the center of many current discussions around the globe. It looms largely in, for example, the Fridays for Future protests, the demands for carbon neutrality, and the concerns about the effects of population aging (Komp-Leukkunen, 2022; Tozer & Klenk, 2018; Wallis & Loy, 2021). Sustainability is defined as a situation in which individuals satisfy their needs without compromising the possibility for future generations to satisfy their needs. This situation has economic, ecological, and social dimensions (Purvis et al., 2019; United Nations, 1987). Because of these diverse dimensions considered, the concept of sustainability can be used to assess a range of current megatrends (Wallenius & Wallenius, 2020).

This study explores how the concept of social sustainability can be used to assess the megatrend of global population aging (Harper, 2019). In 1950, 5% of the world’s population was aged 65 years or older, whereas already 9% was in this age group in 2020. The share is expected to further increase to 16% by 2050 (United Nations, 2019). This demographic change restructures the fabric of societies. It gives more weight to the needs of older individuals, possibly at the costs of younger individuals. Moreover, it challenges, for example, the redistributive mechanisms of pension schemes and the organizational structures of care provisions (Komp & Béland, 2012; Xu et al., 2023). Increasing demands in these areas raise concerns about whether extant social arrangements can be maintained for future generations. Social sustainability denotes a situation in which individuals satisfy their social needs while allowing future generations to do the same. As such, it is one dimension of sustainability, next to the ecological and the economic one (United Nations, 1987). Considering the overlap between the concerns about the effects of population aging and the concept of social sustainability, it seems natural to jointly consider them. The concept of social sustainability can help gerontological research reflect on the desirability and steering possibilities for the effects of population aging. Thereby, it can help researchers set an agenda for the future of aging research that facilitates the welfare and well-being of all current and future generations.

Previous research generated rich buzz, albeit fragmented knowledge, on social sustainability in aging populations (see, e.g., Lorinc et al., 2022; Qvist & Jensen, 2022). Already a look at scholar.google.com shows the increasing popularity of the topic: this search engine returns only five hits for the search terms “social sustainability” and “aging population” for the year 2000, but 50 for 2010, and 225 for 2022! Supplementary Figure 1 displays this trend over time. This article found more than 900 unique texts that mentioned both population aging and social sustainability. This number clearly shows that both concepts are connected in scientific discourse. However, of these texts, only 33 contained a scientific inquiry into both concepts. This low number underlines what several studies already revealed: the concept of social sustainability is multifacetted, underexplored, and lacks a clear definition (Andersson & Gromark, 2021; Boyer et al., 2016; Morozova & Gurova, 2021). Vallance and colleagues (2011) report that it can be used in three fundamentally different ways: as maintaining the status quo, as behavioral modification for achieving environmental goals, or as addressing basic needs. These basic needs encompass, for example, equity, safety, and a high quality of life (Eizenberg & Jabareen, 2017; Vallance et al., 2011). Other researchers highlight that it can refer to processes as well as contexts (Suopajärvi et al., 2016), and that it can be applied to units as different as companies, cities, and politics (Alexander et al., 2020; Davidson, 2009; Hamiduddin, 2015). Hamiduddin concludes that social sustainability is a “work in progress” (2015, p. 29), whereas Vallance and colleagues call it a “concept in chaos” (2011, p. 342). The present article helps to clarify the meaning of social sustainability. It explores its use in a delimited topical area, thereby highlighting a more precise and homogeneous understanding of this context.

This study maps our scientific body of knowledge on the connection between social sustainability and population aging. To do this, it answers three research questions: First, how is social sustainability understood in studies on population aging? The answer to this question lays the foundation for future discussions on social sustainability in aging populations. Second, how does this understanding differ across the topics discussed? This question takes a more nuanced look at the understanding of social sustainability in the context of population aging. It traces how different study topics highlight various facets of the concept of social sustainability. The answer to this question indicates how the varied discussions on social sustainability in aging populations can be integrated. Third, how is population aging connected to social sustainability? This question maps the content-wise knowledge generated by previous studies on the connection between population aging and social sustainability. To answer the research questions, this article presents a systematic literature review, which is a review exploring previous studies in a structured and purposeful way.

The remainder of this article is structured as follows: First, the material analyzed and the method applied are described. Then, the findings relating to each research question are presented. Finally, a discussion section reflects on research challenges and gaps in knowledge that future studies could tackle.

## Research Design and Methods

This article maps the scientific body of knowledge on social sustainability in aging populations. It does this through a systematic literature review, which is an approach used for analytically capturing the state of a research field (Shaffril et al., 2021). It methodically selects, analyzes, and documents literature to answer predefined research questions. In doing so, it traces the literature content and the amount of information available (Petticrew & Roberts, 2008). Therefore, this analytical approach is ideal for young research fields like the one on social sustainability in aging populations.

**Figure 1.**
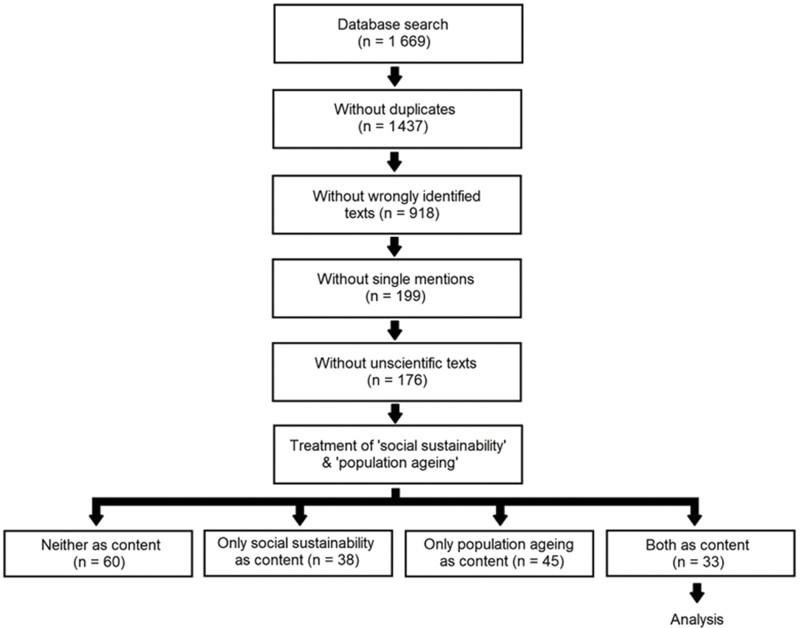
How texts were selected for the analysis.

The literature selection proceeded in six steps (see Figure 1). First, several databases were systematically searched for suitable literature. This search took place in March and April 2022 using the databases scholar.google.com, ProQuest, and JSTOR. These databases were chosen because they cover all scientific disciplines, which is essential in an exploratory study like the present one. Scholar.google.com is the biggest search engine for scientific publications, making it a prime information source. ProQuest specializes in providing information to libraries, and it was included to ensure that all established scientific publications channels are considered. JSTOR specializes in digitalizing older scientific documents, and it was included to ensure that also older publications would be reviewed. Identical searches were carried out in all databases. These searches used all possible combinations of “social sustainability” with one of the following terms: “ageing population*,” “aging population*,” “population ageing,” “population aging,” “ageing societ*,” or “aging societ*.” A list of the search terms and mesh words combinations is presented as Supplementary Material 1. The search rendered a total of 1,669 texts (1,354 from scholar.google.com, 243 from ProQuest, and 72 from JSTOR). Second, duplicates were deleted. After this step, 1,437 unique texts remained. Third, wrongly identified texts were deleted. Texts were considered wrongly identified if they contained the keywords only in the literature list or in the author’s biography but not in the content of the text itself. After this step, 918 texts remained. Fourth, texts were deleted that mentioned at least one keyword only once. These texts usually did this to present them as buzzwords or in enumerations. The content of these keywords was not explored in depth. After this step, 199 texts remained. The high number of deleted texts reveals that many discussions on the combination of social sustainability and population aging remain at a superficial level. These concepts are often jointly used but mainly as buzzwords. Fifth, texts of insufficient scientific quality were deleted. Insufficient scientific quality was defined as not having undergone a peer review. These texts were deleted to ensure that the information analyzed reflected scientific discourse and our current state of knowledge. The omission or passing of a peer-review process was determined by studying the technical information about the texts and the homepages of the publication channels. After this step, 176 texts remained. Sixth, texts were deleted that did not engage with the content of both concepts, social sustainability, and population aging. Such a lack of engagement meant that the texts only described the concepts as part of the social and historical context of the study. They did not explore what the concepts meant. As a result, these texts did not contribute to our understanding of social sustainability in aging populations. After this step, 33 texts remained (see Figure 1; Supplementary Figure 2 shows the PRISMA flow diagram). These remaining texts were analyzed, and they are marked with an asterisk in the reference list. The considerable number of deleted texts in this last step is a testament to the uncertainty about how both concepts relate to each other. Although many researchers acknowledge that social sustainability and population aging coincide, few jointly analyze them in their studies. This situation is typical for a young research field.

All steps of the selection process excluded only those texts that clearly did not address the criterion scanned for. If it could not be conclusively decided whether or not a text met an exclusion criterion, then this text was kept in the sample. This approach kept the number of texts in the initial selection steps higher, funneling more texts into the last selection step where their content was assessed. Thus, this approach ensured that all texts were thoroughly considered and only excluded from the sample once the need for such a step had clearly been asserted. All texts analyzed can be obtained from the journals’ respective publishing houses that published them. Some texts are publicly available free of charge, and others must be paid for.

The texts were analyzed in a thematic analysis, meaning by screening the texts for recurrent themes, which were then grouped under different headings (Shaffril et al., 2021). The thematic analysis was carried out in three steps, with each step corresponding to one research question. First, the definitions of social sustainability were collected from all texts and integrated into one encompassing definition. Second, the texts were grouped according to their topics. Then, the definitions of social sustainability were (a) integrated within the groups and (b) compared across groups. Third, statements about the connections between social sustainability and population aging were collected from all texts. Then, these statements were (a) integrated within the groups and (b) compared across groups. Table 1 shows which understandings and connections were found in each text, presenting the texts grouped according to their topics. If a text did not specify any understanding, then this is marked as such in the table.

**Table 1.** The Understanding of Social Sustainability and Its Connection With Population Aging, by Text and Topic

Topic	Understanding of social sustainability with focus on …			Connection between social sustainability and population aging
	Desirable societies	Quality of life	Current and future generations	
*Topic: Geographical considerations (n = 16)*				
Subtopic: Urban environments (*n* = 10)				
Andersson & Gromark, 2021	x	—	x	In aging populations, health promotion is an important contribution to social sustainability.
Chong et al., 2017	x	x	—	The social sustainability needs to be addressed change as populations age.
Hamiduddin, 2015	x	—	—	Population aging undermines extant infrastructure and services, which reduces social sustainability.
Han et al., 2021	x	x	—	Addressing the needs of the aging population increases social sustainability.
Hu, 2021	x	—	—	Social sustainability perception of old-age housing is more important in aging populations.
Kristl et al., 2020	x	—	—	Population aging changes the needs that need to be considered in social sustainability.
Landorf et al., 2008	x	—	—	Care services, accessibility, and social networks bring social sustainability in aging populations.
Lim et al., 2019	x	—	—	Social sustainability perception of old-age housing is more important in aging populations.
Qian et al., 2019	x	—	—	In aging populations, active aging and health care services are important for social sustainability.
Xie & Batunova, 2020	x	—	x	Population aging weakens the authenticity of ethnic neighborhoods, reducing social sustainability.
Subtopic: Rural areas (*n* = 6)				
Abramsson & Hagberg, 2018	x	—	—	Population aging undermines extant services and social activities, which reduces social sustainability.
Garcia-Casarejos & Saez-Perez, 2020	—	x	—	Population aging necessitates youth migration to rural areas for social sustainability.
Miller et al., 2012	—	—	—	Population aging depletes the workforce in rural areas, thereby lowering social sustainability.
Moren-Alegret et al., 2018	x	—	—	Population aging necessitates youth migration to rural areas for social sustainability.
Nakanishi & Black, 2015	x	—	—	Unsatisfactory public transport in rural areas promotes care driving in old age for social sustainability.
Winterton et al., 2019	x	—	—	Retirement migration to rural areas lowers the social sustainability there.
*Topic: Policymaking (n = 10)*				
Subtopic: Social and health care (*n* = 4)				
Colnar et al., 2019	x	—	—	Aging populations require more social work for social ­sustainability.
Garces et al., 2003	—	—	x	Population aging increases the demand for health care, bringing cutbacks that threaten social sustainability.
Lyons & Duggan, 2015	—	—	—	Population aging can reduce access to health care, which reduces social sustainability.
Woods, 2020	x	—	—	Care technologies for aging populations may reduce social sustainability.
Subtopic: Pension insurance (*n* = 3)				
Ebbinghaus, 2015	x	—	—	Population aging can reduce pension levels, which lowers social sustainability.
Vuckovic & Skuflic, 2021	x	—	—	Population aging can reduce pension levels, which lowers social sustainability.
Zaidi, 2012	x	x	x	Population aging can disadvantage future generations in pension schemes, which lowers social sustainability.
Subtopic: General considerations (*n* = 3)				
Walker, 2021	—	x	—	Pensions need to provide sufficient income to older individuals to achieve social sustainability.
Wurster, 2011	—	—	x	Population aging advances the interests of older individuals at the cost of future generations, which lowers social sustainability. Aging populations bring more knowledge, which brings more socially sustainable policy output.
Zaidi et al., 2012	x	x	x	Policymaking needs to balance the interests of the different generations to achieve social sustainability.
*Topic: The economy and consumption (n = 3)*				
Berlin & Adams, 2017	x	—	—	In aging populations, work opportunities for older individuals are needed for social sustainability.
Farrer, 2022	x	—	—	Aging restaurant owners close down their businesses, which reduces social sustainability.
Morozova & Gurova, 2021	x	—	—	Designing for aging consumers increases social sustainability.
*Topic: Private lives (n = 3)*				
Kumar & Sharma, 2022	x	x	—	Activities in old age bring social sustainability in aging populations.
Wood & Dashper, 2021	x	—	—	Events can facilitate social sustainability in aging populations.
Xia et al., 2014	x	—	—	Population aging makes older people’s knowledge about social sustainability more important.
*Topic: Conceptual considerations (n = 1)*				
Liu et al., 2017	x	x	—	Population aging draws attention to older people’s role in social sustainability.
Total	27	8	6	

*Note*: “*n*” denotes the sample size; “x” stands for “yes”; ‘—‘ stands for “no”.

The literature selection and analysis were carried out in a team of two researchers. Working in a team of two allowed for comparisons of assessments and discussions of unclear points, both of which served as means of quality control. The researchers worked in turns, alternating between carrying out research and reviewing the other researcher’s work. Initially, both researchers decided on the study idea, and the first researcher carried out the preliminary data collection and analysis. Then, both researchers reviewed the literature and findings. The details of the study, literature search, and analysis method were decided. Next, the second researcher carried out a thorough data collection and analysis. The findings were reviewed by both researchers again. Finally, corrections were made where necessary.

To gain a deeper understanding of the part of the discourse that takes place within gerontological journals, a supplementary analysis was carried out. For this analysis, those texts were selected from the original sample, that had been published as articles in SCIMAGO-listed journals in the subject category “gerontology.” Supplementary Material 2 shows which journals were listed in this category as of February 2023. Fourteen texts fell within this category. Next, these 14 texts were traced throughout the steps of text selection. Supplementary Figure 3 summarizes how they fared. After duplicates were removed, 11 unique texts remained. Of these, eight did not contain the keywords in the text itself, but only in, for example, the literature list. Consequently, they were removed. The remaining three texts mentioned at least one keyword only once, for example, in an enumeration or as a buzzword. They did not engage with the content of the keyword. Therefore, also these texts were deleted. As a result, none of the texts qualified for analysis. This pattern of text selection shows that the current discourse on social sustainability in aging populations takes place outside of the gerontology journals listed in SCIMAGO. The articles in these journals draw on relevant texts, but do not engage in depth with their content. This omission leaves room for other disciplines to shape the discourse on social sustainability in aging populations.

This systematic literature review was preregistered with PROSPERO (https://www.crd.york.ac.uk/prospero/display_record.php?RecordID=357016). The PRISMA checklist is included as Supplementary Table 1.

## Results

### The Understanding of Social Sustainability in Aging Populations

**Figure 2.**
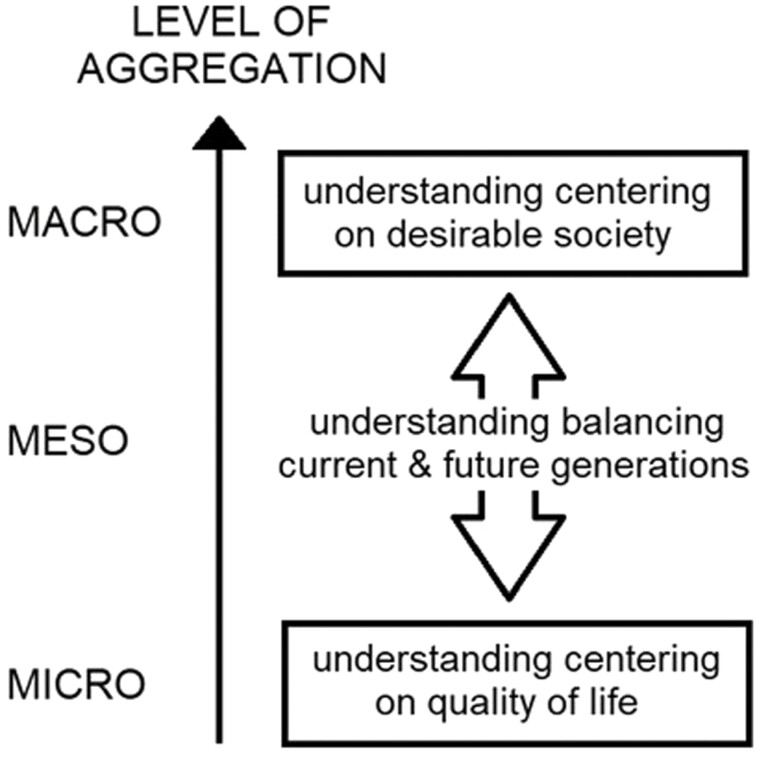
Relationship between the three understandings of social sustainability.

Most studies analyzed discussed their understanding of social sustainability. Only two of the 33 studies did not clarify their understanding of this concept. The other studies outline their understanding, highlighting different ways in which the concept of social sustainability can be interpreted. Overall, three understandings are repeatedly mentioned in the studies. The first one focuses on desirable societies, discussing issues at the macrolevel. The second one centers on questions of the quality of life, focusing on microlevel considerations of individuals. The third understanding explores how to balance the interests of current and future generations, which puts it at the mesolevel and into a supplementary position toward the other two understandings. Figure 2 shows how these understandings are related to one another.

The first understanding of social sustainability centers on desirable societies. It pinpoints desirable qualities that societies need to have to make sustaining them a responsible decision. Abramsson and Hagberg (2018, p. 103) summarize this idea of social sustainability as “functional local societies where people can lead good lives.” Qian and colleagues (2019, p. 659) add that it is “established on the concept that a decision or policy should enhance the betterment of society.” Liu and colleagues (2017, p. 658) hint that social sustainability may not refer to the society as such, but instead to the “essential qualities or goals of societies for long-term development.” Kristl and colleagues (2020, p. 610) choose a different vantage point when suggesting that social sustainability is the “process for creating sustainable and prosperous places that promote well-being.” Despite the disagreement on which part of this complex exactly social sustainability refers to, the message is clear: social sustainability means that extant social structures and processes deserve to be maintained. Twenty-seven of the 33 texts analyzed interpret social sustainability in this way. Consequently, this interpretation is widely shared, although not ubiquitous. However, the texts disagree on what exactly the desirable qualities of societies are that deserve to be maintained.

Broadly speaking, the texts suggest maintaining the qualities of societies that facilitate a good quality of life (Abramsson & Hagberg, 2018; Andersson & Gromark, 2021; Liu et al., 2017), well-being (Colnar et al., 2019; Han et al., 2021; Kristl et al., 2020; Kumar & Sharma, 2022; Liu et al., 2017), and need fulfillment (Han et al., 2021; Liu et al., 2017; Qian et al., 2019; Xia et al., 2014). Several texts argue that these goals can be reached through equality, meaning by treating individuals in the same way (Liu et al., 2017; Morozova & Gurova, 2021; Woods, 2020), or through equity, meaning by treating people in a fair way (Andersson & Gromark, 2021; Berlin & Adams, 2017; Hamiduddin, 2015; Han et al., 2021; Landorf et al., 2008; Liu et al., 2017; Morozova & Gurova, 2021; Nakanashi & Black, 2015; Qian et al., 2019; Vuckovic & Skuflic, 2021; Woods, 2020; Zaidi, 2012; Zaidi et al., 2012). Whether a treatment is fair or not is subjective, depending on the perceptions, preferences, and cultures of the individuals in the respective society (Liu et al., 2017; Vuckovic & Skuflic, 2021). If a treatment is in line with these perceptions, preferences, and culture, then social justice is reached (Han et al., 2021; Kumar & Sharma, 2022; Liu et al., 2017). A few studies add that sustainable societies should also possess basic services and facilities, at least in the areas of health and social care and pensions (Berlin & Adams, 2017; Lim et al., 2019; Moren-Alegret et al., 2018; Qian et al., 2019; Winterton et al., 2019; Zaidi, 2012; Zaidi et al., 2012). Some studies demand that social sustainability pay attention to environmental issues (Abramsson & Hagberg, 2018; Liu et al., 2017; Qian et al., 2019; Winterton et al., 2019; Wood & Dashper, 2021) or financial concerns (Ebbinghaus, 2015; Winterton et al., 2019). These demands link social sustainability back to the other dimensions of sustainability, namely the ecological and economic dimensions. The texts expect social sustainability to affect the fabric of society, enhancing social integration (Liu et al., 2017; Nakanishi & Black, 2015), social cohesion (Liu et al., 2017; Winterton et al., 2019), and participation (Colnar et al., 2019; Farrer, 2022; Landorf et al., 2008; Liu et al., 2017; Morozova & Gurova, 2021; Qian et al., 2019).

The second understanding of social sustainability centers on the quality of life for individuals. It describes what situation is so desirable for individuals that they want to maintain it. Therewith, this individual-level understanding is the pendant to the society-level understanding previously described. Yet only eight out of the 33 studies analyzed subscribe to it. These studies stress that social sustainability is reached if the individuals’ lives have a sufficient quality. Liu and colleagues (2017, p. 658) propose that “social sustainability is about people’s quality of life.” In a similar vein, Walker (2021, p. 161) describes social sustainability for older people as “enable older people to lead lives of decent social quality.” A sufficient quality of life includes need satisfaction, well-being, health, and happiness (Chong et al., 2017; Liu et al., 2017). The individuals should be active (Kumar & Shamar, 2022) and satisfied with their activities (Garcia-Caserejos & Saez-Perez, 2020). Moreover, older individuals should obtain an adequate pension income (Zaidi, 2012; Zaidi et al., 2012).

The third understanding of social sustainability centers on balancing the interests of current and future generations. Wurster (2011, p. 542) summarizes this idea as “the degree of equality of opportunity and participation for future generations.” Zaidi and colleagues (2012, p. 215) conclude that thereby, this approach “includes the intergenerational solidarity aspect.” Garces and colleagues (2003, p. 210) reflect on this understanding from a theoretical perspective, stating that it constitutes “the extension of the welfare principle of Universality in time, in such a way that welfare is a right, not only for the citizens present [ … ], but also for all those people who succeed us in time forming the society of the future.” This understanding of social sustainability demands that current and future generations should have the opportunity to lead good lives, being able to access necessary resources and services (Zaidi, 2012; Zaidi et al., 2012). To achieve this goal, current and future citizens should be included in welfare considerations such as the ones presented in the previous two understandings of social sustainability (Garces et al., 2003). One study suggests that an outcome of this approach would be a decrease in intergenerational conflicts over resources (Zaidi, 2012). Several studies remark that the state of social sustainability is reached when institutions support both current and future generations and when the individuals of both generations have the options and resources to lead good lives (Andersson & Gromark, 2021; Garces et al., 2003; Wurster, 2011; Xie & Batunova, 2020). One study suggests that social sustainability in this understanding is particularly likely to come about when individuals are well-educated and endowed with high human capital (Wurster, 2011). In total, six studies use this understanding of social sustainability.

When the three understandings are jointly considered, a general definition of social sustainability in aging populations can be provided. This definition sees social sustainability as a feature of societies that realizes a high quality of life for its current and future citizens.

### How the Understanding of Social Sustainability Differs Across the Topics Discussed

**Figure 3.**
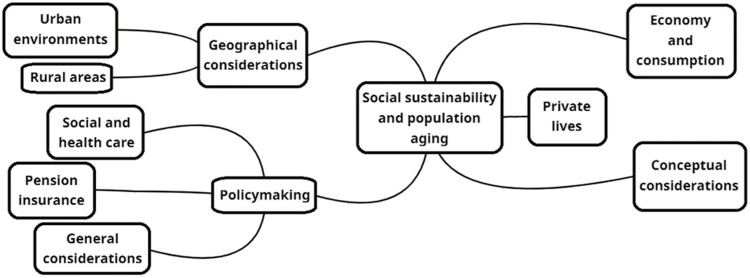
Mind map of the topics discussed in the literature analyzed.

The texts analyzed cover an array of topics, ranging from urban environments to pension insurances. Figure 3 presents a mind map of the topics covered. It shows that discussions cluster around five topics, namely (1) geographical considerations, which encompass discussions on urban environments and rural areas, (2) policymaking, which encompasses discussions on social and health care, pension insurance, and general considerations, (3) economy and consumption, (4) private lives, and (5) conceptual considerations. Although the range of topics is wide, they still cover only a fraction of the discussions on social sustainability in aging populations that we could be having. The understanding of social sustainability used shows some variation with the study topic. Table 1 provides an overview.

The topic most extensively covered is that of geographical considerations, being addressed in 16 texts. Ten of these texts discuss urban environments. They explore urban environments from the level of an individual building (e.g., Hu, 2021; Kristl et al., 2020) to the level of the entire city (e.g., Landorf et al., 2008). All these texts have in common that they use an understanding of social sustainability that focuses on desirable societies. This choice ties in well with the topic of the studies, which likewise focuses on how individuals live together. Four of the texts broaden the discussion by supplementing the understanding by focusing on desirable societies with a second understanding. However, there is no connection between the study topic and the second understanding of social sustainability chosen.

The characteristics of rural areas are reflected in six texts. The aspects of rural areas covered by these texts are very diverse, including migration (Moren-Alegret et al., 2018; Winterton et al., 2019), transport (Nakanishi & Black, 2015), and economic development (Miller et al., 2012), among other things. The diversity of the aspects covered is also reflected in the understanding of social sustainability used. Although four texts use the understanding centering on desirable societies, two do not. One instead uses the understanding centering on the quality of life, whereas the other does not specify its understanding at all. Encompassing approaches that merge two or more understandings do not occur in this group.

The second most common topic is that of policymaking, being discussed in 10 texts. Four of those texts explore social and health care. They study how services must be further developed to become sustainable in times of population aging. The kinds of services considered are healthcare (Garces et al., 2003; Lyons & Duggan, 2015), old-age care (Woods, 2020), and social work (Colnar et al., 2019). Considering the similarity of the perspective adopted in these studies, one might expect them to adopt the same understanding of social sustainability. Yet two chose an understanding focusing on desirable societies, one chose an understanding considering future generations, and the remaining one does not specify its understanding. The difference in understandings can be explained with the different goals of the studies. The texts on social work and old-age care aimed for good quality of the services, and they used the understanding focusing on desirable societies (Colnar et al., 2019; Woods, 2020). In contrast, the text using the understanding considering future generations aimed to develop strategies for future development (Garces et al., 2003). Thus, in this group, the understanding of social sustainability used reflects the goal of the studies more than their topic.

Pension insurance is discussed in three texts. Pension insurance redistributes resources within a society. As such, it lends itself to the understanding of social sustainability that focuses on desirable societies. Thus, it comes as no surprise that all texts exploring pension insurance utilized exactly this understanding (Ebbinghaus, 2015; Vuckovic & Skuflic, 2021; Zaidi, 2012). One of the texts used it together with the other two understandings of social sustainability (Zaidi, 2012).

Moreover, general considerations on policymaking are presented in three texts. These texts take wide perspectives on policymaking, seeking to understand general principles rather than an individual initiative (Walker, 2021; Wurster, 2011; Zaidi et al., 2012). With this approach, the texts touch upon the foci of all three understandings of social sustainability: they explore how to guarantee the well-being of individuals, by means of societal restructuring, while considering present and future generations. Accordingly, the texts also use all three understandings of societal sustainability, with two of them picking one (Walker, 2021; Wurster, 2011) and one merging all three of them (Zaidi et al., 2012).

The third topic is the economy and consumption, which is explored in three texts. These texts look into economic sectors, production, and consumption (Berlin & Adams, 2017; Farrer, 2022; Morozova & Gurova, 2021). Thereby, they tackle phenomena at the societal level. Accordingly, all of them use an understanding of social sustainability that focuses on desirable societies.

The fourth topic is private lives, which is explored in three texts. These texts look into activities, events, and knowledge (Kumar & Sharma, 2022; Wood & Dashper, 2021; Xia et al., 2014). Given the focus on private lives of individuals, one might expect these texts to use the understanding of social sustainability that focuses on quality of life. However, this is not the case. All texts use the understanding focusing on desirable societies, and only one of them combines this understanding with the one focusing on quality of life. This situation reveals that even though the texts explored a topic relating to individuals, they did this from a societal perspective.

The final topic is conceptual considerations. Only one text took this approach, reflecting on the meaning of social sustainability in China (Liu et al., 2017). It argues that the understanding of social sustainability is culture-specific because the philosophical ideologies underlying the concept of social sustainability are culture-specific. The text arrived at this conclusion after considering social sustainability in the understandings focusing on desirable societies and on quality of life.

### How Population Aging and Social Sustainability Are Connected

The multiple facets of social sustainability give it diverse connections to population aging. These connections roughly align with the topics discussed, as Table 1 highlights. The discussion of geographical considerations stresses that the means for achieving social sustainability change as populations age. The texts studying urban environments outline how these environments change when older individuals age in place (e.g., Chong et al., 2017). Chong et al. (2017), Kristl et al. (2020), and Han et al. (2021) point out that population aging brings about a change in which needs have to be addressed if social sustainability is to be achieved. Kristl et al. (2020) make this argument referring only to the understanding of social sustainability focusing on desirable societies, whereas Chong et al. (2017) and Han et al. (2021) additionally consider the older individuals’ quality of life. Hamiduddin (2015) underlines that when adaptations are foregone, population aging undermines extant infrastructures and services. He uses the understanding of social sustainability as the desirability of a society. Qian et al. (2019) and Landorf et al. (2008) suggest changing especially health care services, active aging options, accessibility features, and social networks. When doing so, they understand social sustainability as a desirable quality of society. Andersson and Gromark (2021) additionally consider the rights of future generations, suggesting a broad focus on health promotion instead of a narrowing one on health care. Xie and Batunova (2020) worry that the changes brought about by population aging may dilute ethnic neighborhoods, making them less socially sustainable. They explain that these neighborhoods would become less desirable, and that future generations may no longer be able to experience them. Lim et al. (2019) and Hu (2021) adopt the perspective of desirable societies, stressing that social sustainability discourses need to increasingly reflect on old-age housing as populations age.

The texts on rural areas agree that the social sustainability of rural areas changes when populations age. Miller et al. (2012) stressed that population aging depletes the workforce in rural areas, which lowers the social sustainability of these areas. They do not specify which understanding of social sustainability brought them to this conclusion. Moren-Alegret et al. (2018) and Garcia-Casarejos and Saez-Perez (2020) agree that youth migration to rural areas would be necessary to counter this trend. Moren-Alegret et al. (2018) thought of desirable societies, whereas Garcia-Casarejos and Saez-Perez (2020) thought of the individuals’ quality of life when making this statement. Winterton and colleagues (2019) add that from a societal perspective, retirement migration to rural areas aggravates the situation. Abramsson and Hagberg (2018) observe that population aging undermines services and social activities in rural areas, lowering the social sustainability of these rural societies. Nakanishi and Black (2015) provide the example of unsatisfactory public transport options for older individuals, which push them to utilize private cars until a high age.

The discussions on policymaking reflect on emerging challenges as well as solutions. The texts on social and health care agree that aging populations require more health care and social work (Colnar et al., 2019; Garces et al., 2003; Lyons & Duggan, 2015; Woods, 2020). Colnar and colleagues (2019) argue that an increased social care provision is needed to maintain the societal qualities needed for social sustainability. Garces et al. (2003) fear that cutbacks in health care may result, which would infringe on the rights of future generations. Lyons and Duggan (2015) share the concerns about cutbacks, fearing for social sustainability in general without specifying how they understand this concept. Woods (2020) remarks that care technologies replacing human interaction may not be a solution to this dilemma. This replacement may render societies less desirable, thereby reducing social sustainability.

The texts on pension insurance add that pension levels may be reduced in reaction to population aging, which would make societies less desirable (Ebbinghaus, 2015; Vuckovic and Skuflic, 2021). Zaidi (2012) takes a wider perspective on this possibility, stressing that it would also affect the rights of future generations.

The texts presenting general considerations on policymaking stress that pension levels have to be sufficient (Walker, 2021) and the rights of all generations have to be considered (Zaidi et al., 2012) if social sustainability is to be achieved. Walker (2021) reached his conclusion when pondering the quality of life, whereas Zaidi et al. (2012) reached their conclusion when considering all understandings of social sustainability. Wurster (2011) makes a more detailed assessment, pondering the rights of future generations. He points out that the growing number of older individuals gives them an advantage over younger generations in organized interests. At the same time, the higher knowledge of the older individuals involved in policymaking and political discussions can generate more socially sustainable policy outputs.

The texts on the economy and consumption take a more solution-oriented approach while reflecting from a societal perspective. Farrer (2022) and Berlin and Adams (2017) reflect on the aging workforce. Farrer (2022) remarks that social sustainability drops when aging restaurant owners close down their businesses. Berlin and Adams (2017) suggest providing more work opportunities for older individuals to maintain the workforce size and social sustainability. Morozova and Gurova (2021) point out that addressing the aging population as consumers may even create business opportunities, which increase social sustainability.

The texts on private lives point out that participating in events and activities in old age can facilitate social sustainability in aging populations (Kumar & Sharma, 2022; Wood & Dashper, 2021). Kumar and Sharma (2022) reached this conclusion when considering desirable societies and the quality of life, whereas Wood and Dashper (2021) considered only societies. Xia and colleagues (2014) add that from a societal perspective, the older individuals’ knowledge on social sustainability becomes more relevant as populations age.

The text presenting conceptual considerations sums the discussion up with the insight that the role of older individuals in social sustainability matters is of increasing importance as populations age (Liu et al., 2017). It came to this insight when considering the desirability of societies and the individuals’ quality of life.

## Discussion and Implications

Social sustainability is one of the key buzzwords in current public and political discussions. It is considered a useful and constructive approach for dealing with megatrends in society, such as population aging. It adds to our scientific body of knowledge by reflecting on how desirable the societal effects of population are. Thereby, it opens them up for steering efforts. The scientific discourse on social sustainability in aging populations is still emerging, being hampered by a fragmented and multifacetted understanding of social sustainability. This article contributes to developing the discourse by clarifying the concept of social sustainability as it is used in studies on population aging.

The first research question is how social sustainability is understood in studies on population aging. Findings show that three different understandings are used side by side: one focusing on desirable societies, one focusing on quality of life, and one including current and future generations. The understanding focusing on desirable societies is most commonly used. Yet the other two understandings also occur individually or in combination with any other understanding. As a result, the suggestion of previous studies that social sustainability lacks a clear definition needs to be corrected (Andersson & Gromark, 2021; Morozova & Gurova, 2021). We have not one but three clear definitions of social sustainability. The inconsistencies in the discourse on social sustainability do not stem from a lack of definitional clarity but from the use of alternative understandings. Future research could help to consolidate social sustainability discourse by always explicitly stating which understanding they subscribe to. Alternatively, they could define social sustainability more generally as a feature of societies that realizes a high quality of life for its current and future citizens.

The second research question is how the understanding of social sustainability differs across the topics discussed. Findings show that the understanding of social sustainability chosen partly aligns with the study topic. However, it also depends on the goal of the study and on the perspective applied. This insight suggests that there is a practical reason for having three different understandings of social sustainability: it reflects the diversity of the discussion held. Using three understandings allows the scientific discussion to explore more topics, adopt more perspectives, and pursue more goals. Therefore, it seems advisable to keep on utilizing all three understandings of social sustainability to build an encompassing body of scientific knowledge.

The third research question is how population aging is connected to social sustainability. Findings show an array of connections. Many texts describe challenges to social sustainability, especially in the areas of pensions, social and health care, and in urban and rural areas. A few texts see opportunities for social sustainability, especially when it comes to the economy, consumption, and policymaking. Some texts recommend a different approach to social sustainability, especially in the areas of urban planning, policymaking, and research. The connection described depends more strongly on the area considered than on the understanding of social sustainability used. Interestingly, the conclusions of studies harmonized with one another, even when different understandings were used. This insight suggests that the three understandings of social sustainability used highlight different aspects of the same underlying phenomenon.

This study has scientific and societal implications. Scientific implications arise because this study narrows down the wide and varied understanding of social sustainability for the context of population aging. It shows that in discussions on population aging, only one of the aspects of social sustainability according to Vallance and colleagues (2011) is used: that of addressing basic needs. However, the heterogeneity in terms of the units considered and of the question of processes versus contexts is not resolved (Alexander et al., 2020; Davidson, 2009; Suopajärvi et al., 2016). Knowledge on the more homogenous understanding of social sustainability in studies on population aging shows how researchers view population aging. It suggests they considered it a matter of fulfilling basic needs, but not of maintaining the status quo or of finding means for achieving environmental goals (compare Vallance et al., 2011). Equipped with this insight, the extant research on social sustainability in aging populations can be combined more easily. Moreover, researchers wishing to join the discourse can do so with a greater certainty on how to interpret extant statements and findings. Especially researchers in gerontology can benefit from this insight. The gerontological contribution to discussions on social sustainability in aging populations is still in its early stages, despite its obvious relevance for these discussions. Researchers in gerontology can use the present article to develop an understanding of how their research fits in with the current state of knowledge, and to decide which understanding of social sustainability they wish to utilize. As a result, they can more easily contribute to cumulative knowledge building in this area.

Societal implications arise because this study can help policymakers and practitioners better understand scientific discussions on social sustainability in aging populations. It shows what the discussions may be referring to when talking about social sustainability, and it can explain why some discussion contributions seem to be at odds with each other. Equipped with this additional knowledge, policymakers and practitioners can more easily introduce research-based social sustainability measures into their work.

Despite its merits, this study also has some limitations. First, it analyzed scientific texts only. Scientific texts may understand social sustainability in a different way than public discourses. Therefore, the findings cannot simply be transferred to public discourses. Instead, a separate follow-up study is needed on public discourses on social sustainability in aging populations. Second, this study analyzes scientific publications written in English. It is possible that the scientific discourses in languages other than English differ from the one in English. A follow-up study should test for such possible differences.

All in all, this study documents the wide academic awareness of the topic of social sustainability in aging populations. This wide awareness goes hand in hand with only a few studies explicitly exploring this topic. The extant studies use three different understandings of social sustainability, which can lead to misunderstandings when combining the insight from the different studies. Therefore, it is essential that future research on social sustainability in aging populations clearly states which of the understandings it subscribes to. This study can help researchers make an informed decision on which understanding to adopt.

## Supplementary Material

gnad097_suppl_Supplementary_Material

## Data Availability

The study materials and analytic approach are available to other researchers for replication. The study materials can be obtained from the journals and publishing houses that published the article’s respective books and book chapters. The analytic approach is described in this article and in PROSPERO, where this study was preregistered (ID CRD42022357016). The search terms and MESH words combinations are listed in Supplementary Material 1.
