# Supplementary material for: Social Sustainability in Aging Populations: A Systematic Literature Review
Source: Gerontologist. 2023 Aug 1;64(5):gnad097. doi: 10.1093/geront/gnad097 (PMC11036160; doi:10.1093/geront/gnad097)
Supplement: gnad097_suppl_Supplementary_Material [file gnad097_suppl_supplementary_material.docx]

**Online Supplementary Material**

Supplementary Figure 1: Number of publications including the search terms “social sustainability” and “aging population”, by year (scholar.google.com, 11.02.2023)


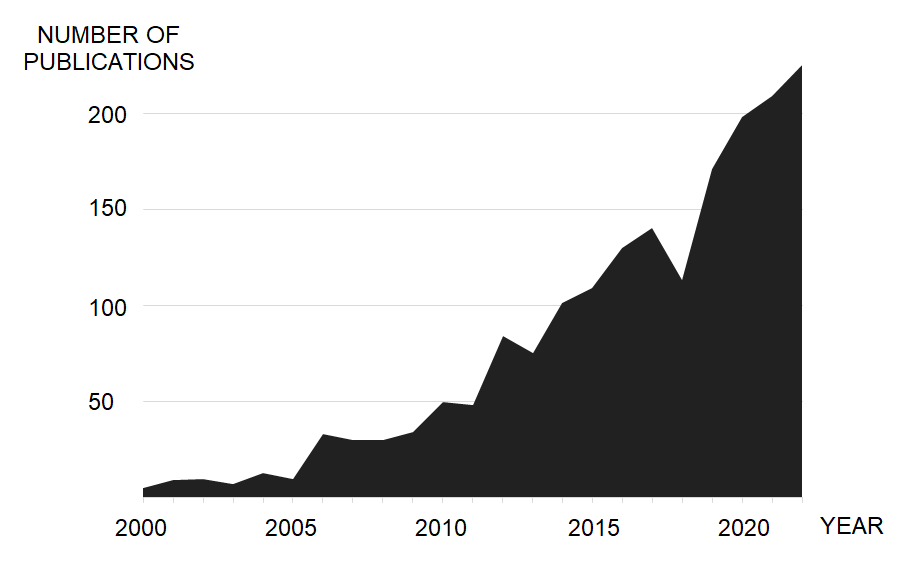


Supplementary Material 1: Search terms and mesh words combinations

The search used all possible combinations of “social sustainability” with one of the following terms: “ageing population*,” “aging population*,” “population ageing,” “population aging,” “ageing societ*,” or “aging societ*.”

The mesh words combinations used for ProQuest and JSTOR are:

- “social sustainability” AND “ageing population*”
- “social sustainability” AND “aging population*”
- “social sustainability” AND “population ageing”
- “social sustainability” AND “population aging”
- “social sustainability” AND “ageing societ*”
- “social sustainability” AND “aging societ*”

The mesh words combinations for scholar.google.com had to be modified because this search engine rendered a much higher number of publications than the other two databases. Therefore, the mesh words combinations were optimized to reduce the number of duplicate findings rendered. The mesh words combinations used were:

- “social sustainability” AND “ageing population*”
- “social sustainability” AND “aging population*” NOT “ageing population*”
- “social sustainability” AND “population ageing” NOT “aging population*” NOT “ageing population”
- “social sustainability” AND “population aging” NOT “population ageing” NOT “aging population*” NOT “ageing population”
- “social sustainability” AND “ageing societ*” NOT “population aging” NOT “population ageing” NOT “aging population*” NOT “ageing population”
- “social sustainability” AND “aging societ” NOT “ageing societ*” NOT “population aging” NOT “population ageing” NOT “aging population*” NOT “ageing population”

Supplementary Figure 2: PRISMA 2020 flow diagram for new systematic reviews which included searches of databases and registers only

Records identified from*:

Databases (n = 3)

Registers (n = 0)

Records removed *before screening*:

Duplicate records removed (n = 232)

Records marked as ineligible by automation tools (n = 0)

Records removed for other reasons (n = 0)

Records screened

(n = 1437)

Records excluded**

(n = 1261)

Reports sought for retrieval

(n = 176)

Reports not retrieved

(n = 0)

Reports assessed for eligibility

(n = 176)

Reports excluded:

Reason 1 (n = 60)

Reason 2 (n = 38)

Reason 3 (n = 45)

Studies included in review

(n = 33)

Reports of included studies

(n = 33)

**Identification of studies via databases and registers**

**Identification**

**Screening**

**Included**

Supplementary Material 2: Journals listed in SCIMAGO in the subject category “gerontology” (February 2023)

- The Gerontologist
- Journal of Aging and Social Policy
- Journals of Gerontology - Series B Psychological Sciences and Social Sciences
- International Psychogeriatrics
- Aging and Mental Health
- Journal of Applied Gerontology
- Archives of Gerontology and Geriatrics
- Journal of Aging and Health
- JMIR Aging
- Alzheimer Disease and Associated Disorders
- Biogerontology
- Geriatrics and Gerontology International
- Canadian Geriatrics Journal
- Clinical Gerontologist
- European Geriatric Medicine
- International journal of older people nursing
- Geriatric Nursing
- Geriatrics (Switzerland)
- Canadian Journal on Aging
- Psychogeriatrics
- International Journal of Qualitative Studies on Health and Well-being
- Journal of Aging and Physical Activity
- Research in gerontological nursing
- GeroPsych: The Journal of Gerontopsychology and Geriatric Psychiatry
- African Journal of Emergency Medicine
- Journal of Gerontological Nursing
- Journal of Aging and Environment
- Activities, Adaptation and Aging
- Zeitschrift fur Gerontologie und Geriatrie
- Quality in Ageing and Older Adults
- Physical and Occupational Therapy in Geriatrics
- Working with Older People
- Nursing older people
- Gerokomos
- Journal of Korean Gerontological Nursing
- Bereavement Care
- Gerontechnology
- Tijdschrift voor Gerontologie en Geriatrie
- Gerontologie et Societe
- Advances in Gerontology
- Soins Gerontologie
- Annals of Long-Term Care
- Annual Review of Gerontology and Geriatrics

Supplementary Figure 3: How texts from SCIMAGO-listed gerontology journals were selected for the analysis


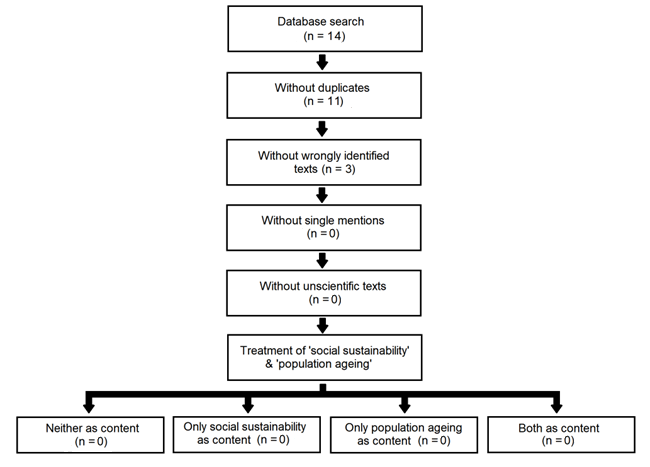


Supplementary Table 1: PRISMA 2020 Checklist

| **Section and Topic** | **Item #** | **Checklist item** | **Location where item is reported** |
| --- | --- | --- | --- |
| **TITLE** | | |  |
| Title | 1 | Identify the report as a systematic review. | title |
| **ABSTRACT** | | |  |
| Abstract | 2 | See the PRISMA 2020 for Abstracts checklist. | abstract |
| **INTRODUCTION** | | |  |
| Rationale | 3 | Describe the rationale for the review in the context of existing knowledge. | “Background and objectives” section |
| Objectives | 4 | Provide an explicit statement of the objective(s) or question(s) the review addresses. | “Background and objectives” section |
| **METHODS** | | |  |
| Eligibility criteria | 5 | Specify the inclusion and exclusion criteria for the review and how studies were grouped for the syntheses. | “Research design and methods” section |
| Information sources | 6 | Specify all databases, registers, websites, organisations, reference lists and other sources searched or consulted to identify studies. Specify the date when each source was last searched or consulted. | “Research design and methods” section |
| Search strategy | 7 | Present the full search strategies for all databases, registers and websites, including any filters and limits used. | “Research design and methods” section |
| Selection process | 8 | Specify the methods used to decide whether a study met the inclusion criteria of the review, including how many reviewers screened each record and each report retrieved, whether they worked independently, and if applicable, details of automation tools used in the process. | “Research design and methods” section |
| Data collection process | 9 | Specify the methods used to collect data from reports, including how many reviewers collected data from each report, whether they worked independently, any processes for obtaining or confirming data from study investigators, and if applicable, details of automation tools used in the process. | “Research design and methods” section |
| Data items | 10a | List and define all outcomes for which data were sought. Specify whether all results that were compatible with each outcome domain in each study were sought (e.g. for all measures, time points, analyses), and if not, the methods used to decide which results to collect. | “Research design and methods” section |
|  | 10b | List and define all other variables for which data were sought (e.g. participant and intervention characteristics, funding sources). Describe any assumptions made about any missing or unclear information. | does not apply |
| Study risk of bias assessment | 11 | Specify the methods used to assess risk of bias in the included studies, including details of the tool(s) used, how many reviewers assessed each study and whether they worked independently, and if applicable, details of automation tools used in the process. | does not apply |
| Effect measures | 12 | Specify for each outcome the effect measure(s) (e.g. risk ratio, mean difference) used in the synthesis or presentation of results. | does not apply |
| Synthesis methods | 13a | Describe the processes used to decide which studies were eligible for each synthesis (e.g. tabulating the study intervention characteristics and comparing against the planned groups for each synthesis (item #5)). | does not apply |
|  | 13b | Describe any methods required to prepare the data for presentation or synthesis, such as handling of missing summary statistics, or data conversions. | does not apply |
|  | 13c | Describe any methods used to tabulate or visually display results of individual studies and syntheses. | “Research design and methods” section |
|  | 13d | Describe any methods used to synthesize results and provide a rationale for the choice(s). If meta-analysis was performed, describe the model(s), method(s) to identify the presence and extent of statistical heterogeneity, and software package(s) used. | “Research design and methods” section |
|  | 13e | Describe any methods used to explore possible causes of heterogeneity among study results (e.g. subgroup analysis, meta-regression). | does not apply |
|  | 13f | Describe any sensitivity analyses conducted to assess robustness of the synthesized results. | does not apply |
| Reporting bias assessment | 14 | Describe any methods used to assess risk of bias due to missing results in a synthesis (arising from reporting biases). | does not apply |
| Certainty assessment | 15 | Describe any methods used to assess certainty (or confidence) in the body of evidence for an outcome. | does not apply |
| **RESULTS** | | |  |
| Study selection | 16a | Describe the results of the search and selection process, from the number of records identified in the search to the number of studies included in the review, ideally using a flow diagram. | Figure 1 and “Research design and methods” section |
|  | 16b | Cite studies that might appear to meet the inclusion criteria, but which were excluded, and explain why they were excluded. | does not apply; see “Research design and methods” section for an explanation |
| Study characteristics | 17 | Cite each included study and present its characteristics. | Table 1 |
| Risk of bias in studies | 18 | Present assessments of risk of bias for each included study. | does not apply |
| Results of individual studies | 19 | For all outcomes, present, for each study: (a) summary statistics for each group (where appropriate) and (b) an effect estimate and its precision (e.g. confidence/credible interval), ideally using structured tables or plots. | does not apply |
| Results of syntheses | 20a | For each synthesis, briefly summarise the characteristics and risk of bias among contributing studies. | does not apply |
|  | 20b | Present results of all statistical syntheses conducted. If meta-analysis was done, present for each the summary estimate and its precision (e.g. confidence/credible interval) and measures of statistical heterogeneity. If comparing groups, describe the direction of the effect. | does not apply |
|  | 20c | Present results of all investigations of possible causes of heterogeneity among study results. | does not apply |
|  | 20d | Present results of all sensitivity analyses conducted to assess the robustness of the synthesized results. | does not apply |
| Reporting biases | 21 | Present assessments of risk of bias due to missing results (arising from reporting biases) for each synthesis assessed. | does not apply |
| Certainty of evidence | 22 | Present assessments of certainty (or confidence) in the body of evidence for each outcome assessed. | does not apply |
| **DISCUSSION** | | |  |
| Discussion | 23a | Provide a general interpretation of the results in the context of other evidence. | “Discussion and implications” section |
|  | 23b | Discuss any limitations of the evidence included in the review. | “Discussion and implications” section |
|  | 23c | Discuss any limitations of the review processes used. | “Discussion and implications” section |
|  | 23d | Discuss implications of the results for practice, policy, and future research. | “Discussion and implications” section |
| **OTHER INFORMATION** | | |  |
| Registration and protocol | 24a | Provide registration information for the review, including register name and registration number, or state that the review was not registered. | “Research design and methods” section and acknowledgements |
|  | 24b | Indicate where the review protocol can be accessed, or state that a protocol was not prepared. | “Research design and methods” section and acknowledgements |
|  | 24c | Describe and explain any amendments to information provided at registration or in the protocol. | “Research design and methods” section and acknowledgements |
| Support | 25 | Describe sources of financial or non-financial support for the review, and the role of the funders or sponsors in the review. | Funding statement |
| Competing interests | 26 | Declare any competing interests of review authors. | Conflict of interest statement |
| Availability of data, code and other materials | 27 | Report which of the following are publicly available and where they can be found: template data collection forms; data extracted from included studies; data used for all analyses; analytic code; any other materials used in the review. | “Research design and methods” section |

*From:*  Page MJ, McKenzie JE, Bossuyt PM, Boutron I, Hoffmann TC, Mulrow CD, et al. The PRISMA 2020 statement: an updated guideline for reporting systematic reviews. BMJ 2021;372:n71. doi: 10.1136/bmj.n71

For more information, visit: <http://www.prisma-statement.org/>
